# Do Perfluoroalkyl Compounds Impair Human Semen Quality?

**DOI:** 10.1289/ehp.0800517

**Published:** 2009-03-02

**Authors:** Ulla Nordström Joensen, Rossana Bossi, Henrik Leffers, Allan Astrup Jensen, Niels E. Skakkebæk, Niels Jørgensen

**Affiliations:** 1 University Department of Growth and Reproduction, Rigshospitalet, Copenhagen, Denmark; 2 National Environmental Research Institute, University of Aarhus, Roskilde, Denmark; 3 FORCE Technology, Brøndby, Denmark

**Keywords:** endocrine disruptors, male reproductive health, perfluoroalkyl compounds, PFAA, PFC, semen quality, sperm morphology, testosterone

## Abstract

**Background:**

Perfluoroalkyl acids (PFAAs) are found globally in wildlife and humans and are suspected to act as endocrine disruptors. There are no previous reports of PFAA levels in adult men from Denmark or of a possible association between semen quality and PFAA exposure.

**Objectives:**

We investigated possible associations between PFAAs and testicular function. We hypothesized that higher PFAA levels would be associated with lower semen quality and lower testosterone levels.

**Methods:**

We analyzed serum samples for levels of 10 different PFAAs and reproductive hormones and assessed semen quality in 105 Danish men from the general population (median age, 19 years).

**Results:**

Considerable levels of perfluorooctane sulfonic acid (PFOS), perfluorooctanoic acid (PFOA), and perfluorohexane sulfonic acid were found in all young men (medians of 24.5, 4.9, and 6.6 ng/mL, respectively). Men with high combined levels of PFOS and PFOA had a median of 6.2 million normal spermatozoa in their ejaculate in contrast to 15.5 million among men with low PFOS–PFOA (*p* = 0.030). In addition, we found nonsignificant trends with regard to lower sperm concentration, lower total sperm counts, and altered pituitary–gonadal hormones among men with high PFOS–PFOA levels.

**Conclusion:**

High PFAA levels were associated with fewer normal sperm. Thus, high levels of PFAAs may contribute to the otherwise unexplained low semen quality often seen in young men. However, our findings need to be corroborated in larger studies.

Perfluoroalkyl acids (PFAAs) are degradation products of many man-made polyfluorinated compounds (PFCs) used in consumer and industrial products, for example, for impregnation of carpets, textiles, and paper (Jensen et al. 2008; Jensen and Leffers 2008; Kissa 2001). Studies of environment, wildlife, and humans suggest widespread presence and exposure, persistence in the environment, and bioaccumulation (Giesy and Kannan 2001; Kannan et al. 2004). For perfluoro octanoic acid (PFOA), perfluorooctane sulfonic acid (PFOS), and perfluorohexane sulfonic acid (PFHxS), three of the most abundant PFAAs, half-lives for humans have been estimated as 3.8, 5.4, and 8.5 years, respectively (Olsen et al. 2007). Some studies suggest that men may have higher serum concentrations of PFAAs than women, and younger individuals may have higher levels than older (Calafat et al. 2006). Thus, young men may have particularly high levels of exposure and may therefore be a group at risk for potential adverse effects of PFAAs.

PFAAs can cross the placental barrier and therefore have the potential to affect the fetus. In humans, levels of PFOS and PFOA in umbilical cord blood have been inversely related to birth weight (Apelberg et al. 2007). In addition, PFOS, PFOA, and PFHxS have been detected in human seminal plasma samples (Guruge et al. 2005). However, data on effects in humans are scarce, and most come from studies of occupationally exposed individuals. These studies have not given conclusive evidence of adverse effects. In a recent study, however, Fei et al. (2009) measured PFAA levels in early pregnancy and found that higher levels of PFOS and PFOA were associated with significantly longer waiting time to pregnancy.

Animal studies provide some evidence for adverse reproductive effects on animals exposed as adults or *in utero*. Exposure of adult male rats to PFOA reduced their testosterone levels and increased their estradiol levels, which may partly explain earlier findings of induction of Leydig cell hyperplasia and/or adenomas in the testes of exposed animals (Biegel et al. 1995; Cook et al. 1992).

Our objective in the present study was to investigate the associations between PFOS, PFOA, PFHxS, and other PFAAs and testicular function. Our primary hypothesis was that high concentrations of PFAAs would be associated with low testosterone levels and that high PFAA levels are negatively associated with semen quality variables.

## Materials and Methods

### Study population

Since 1996, semen quality of young men in Denmark has been surveyed in a cross-sectional study (Andersen et al. 2000; Jørgensen et al. 2002). All young Danish men must report for military draft, and annually new cohorts of approximately 300 men from the Copenhagen area have been included. They each provided one semen sample and had a venous blood sample drawn. Of the 546 men examined in 2003, we selected 105 for the investigation of association between PFAAs and testicular function. The 105 men included the 53 men (group 1) with the highest testosterone levels (median, 31.8 nmol/L; range, 30.1–34.8 nmol/L) and the 52 men (group 2) with the lowest testosterone levels (median, 14.0 nmol/L; range, 10.5–15.5 nmol/L). We chose the men examined in 2003 because this was the latest year from which we had completed analyses of reproductive hormones. The median ages of men were as follows: for group 1, 18.9 years (range, 18.2–24.6); for group 2, 19.0 years (range, 18.2–25.2); and for all 105 young men, 19.0 years (range, 18.2–25.2). Information on ejaculation abstinence period and hour of blood sampling was recorded. All samples of semen and blood were collected between 0830 and 1315 hours. Serum was stored at −20°C until chemical analysis.

The Danish National Committee on Biomedical Research Ethics, Copenhagen Region, approved the research, and all young men gave written informed consent.

### PFAA analysis

Thawed serum samples were analyzed in January 2008 for 10 different perfluorinated chemicals with carbon chain length from C6 to C13: PFHxS, perfluoroheptanoic acid (PFHpA), PFOA, PFOS, perfluorooctane sulfonamide (PFOSA), perfluoro nonanoic acid (PFNA), perfluorodecanoic acid (PFDA), perfluoroundecanoic acid (PFUnA), perfluoro dodecanoic acid (PFDoA), and perfluorotridecanoic acid (PFTrA). One milliliter of serum was spiked with the surrogate standards ^13^C_8_-PFOA, ^13^C_2_-PFDA, and 13C_4_-PFOS and extracted according to the ion pairing method described previously (Hansen et al. 2001). Matrix-matched standards were prepared by spiking rabbit serum (Sigma Aldrich, Schnelldorf, Germany) with the analytes and the surrogate standards. Blank samples consisted of rabbit serum spiked with only surrogate standards. Standards and blanks were extracted together with each batch of samples.

Instrumental analysis was performed by liquid chromatography-tandem mass spectrometry (LC-MS-MS) with electrospray ionization. The extracts (20 μL injection volume) were chromatographed on a C18 Betasil column (2.1 × 50 mm; Thermo Hypesil-Keystone, Bellefonte, PA, USA) using an Agilent 1100 Series HPLC (Agilent Technologies, Palo Alto, CA, USA). The HPLC was interfaced to a triple quadrupole API 2000 (Sciex, Concorde, Ontario, Canada) equipped with a TurboIon Spray source operating in negative ion mode. Chromatographic conditions and transition MS-MS ions have been described in detail previously (Bossi et al. 2005). The limits of detection (LODs) ranged from 0.1 to 0.5 ng/mL.

### Reproductive hormone analysis

We analyzed thawed serum samples for the levels of testosterone, estradiol, sex hormone binding globulin (SHBG), luteinizing hormone (LH), follicle-stimulating hormone (FSH), and inhibin B, as described previously (Paasch et al. 2008). Free androgen index (FAI) was calculated as (testosterone × 100/SHBG). Ratios between hormones were calculated by simple division.

### Semen analysis

We assessed semen volume by weight and determined the sperm concentration using a Bürker-Türk hemocytometer (Paul Marienfeld GmbH & Co. KG, Lauda-Königshofen, Germany). We calculated total sperm count as semen volume × sperm concentration. The percentage of motile spermatozoa [World Health Organization (WHO) class A + B + C (WHO 1999)] was assessed on fresh samples. Sperm morphology slides were fixed and Papanicolaou-stained, and all were assessed according to strict criteria (Menkveld et al. 1990) by one trained technician over a period of 1 week. The method for semen analysis has been described in detail previously (Jørgensen et al. 2002).

### Statistical analysis

We used medians and 5–95th percentiles to describe the levels of PFAAs in groups 1 and 2. The Mann-Whitney *U*-test was used to compare groups 1 and 2 with respect to PFAA levels, body mass index (BMI), and smoking status. Samples with values < LOD were set to 0 ng/mL. We used Pearson’s correlation coefficients and related *p*-values to describe correlations between levels of different PFCs. Univariate regression analysis was performed for comparison of hormone levels between groups 1 and 2 and to describe associations between PFAAs and hormones or semen variables. Sperm concentration, semen volume, and total sperm count were adjusted for the effect of ejaculation abstinence period. Sperm motility was adjusted for time between ejaculation and assessment of motility. Sex hormone concentrations were adjusted for hour of blood sampling. Semen variables and hormone levels and ratios, except sperm morphology and total testosterone, were ln-transformed to obtain normality of the residuals. Smoking and BMI were tested for confounding effects but, because neither was significant, we did not include them in the final analyses.

We analyzed for associations between PFAAs and testicular function (hormone levels and semen quality) for the whole group of 105 men. We singled out PFOS and PFOA, and calculated results as estimated changes in end point (reproductive hormones and semen variables) with a change in serum concentration of 1 ng/mL PFOS, PFOA, or the summed concentration of PFOS plus PFOA. We divided the men into three groups from the combined concentrations of PFOS and PFOA. Each sample was given a quartile score of 1–4 for PFOS and PFOA levels separately. A score of 1 was assigned to samples with levels within the lowest quartile, and a score of score 4 was assigned to those with concentrations within the highest quartile. We then summed the quartile scores for PFOS and PFOA, giving each sample a possible score of 2–8. Samples were then divided into three quartile groups for PFOS and PFOA combined: low-PFAA group (*n* = 29) with summed quartile score of 2–3, intermediate-PFAA group (*n* = 48) with score of 4–6, and high-PFAA group (*n* = 28) with score of 7–8. Analysis for association between quartile group and hormone levels or semen variables was conducted using univariate regression analysis, adjusted for the above-mentioned confounders.

We performed statistical analysis using SPSS statistical software, version 16.0 (SPSS Inc., Chicago, IL, USA).

## Results

### Levels of PFAAs in serum

The serum levels and number of samples > LOD for all PFAAs are shown in Table 1 for the low and high testosterone groups and for all 105 men. Except for PFOSA, which was detected in only 56 men, we found no significant difference in levels of any PFAA between groups 1 and 2. The median PFOS, PFOA, and PFHxS concentrations for all 105 of the men were 24.5, 4.9, and 6.6 ng/mL, respectively, and only these were included in the final regression analyses. The remaining PFAAs were found in much lower concentrations; therefore, these results are not discussed further. PFOS levels were positively correlated with concentrations of PFOA (*r* = 0.594, *p* < 0.0005) and PFHxS (*r* = 0.304, *p* = 0.002). PFOA and PFHxS levels were positively correlated but not statistically significantly (*r* = 0.136, *p*= 0.2).

### PFAAs and semen variables

**Figure 1:**
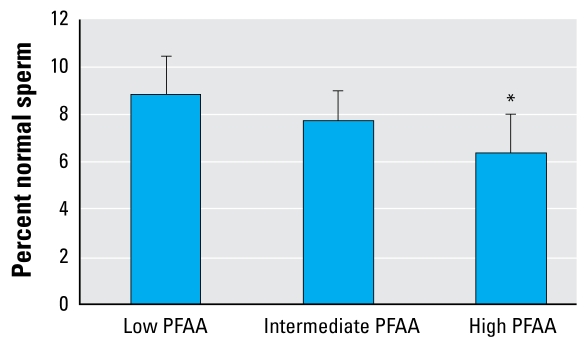
Percent morphologically normal spermatozoa by PFAA quartile group (adjusted mean and 95% confidence interval) in all 105 men. *p= 0.037 compared with low PFAA quartile group.

**Figure 2:**
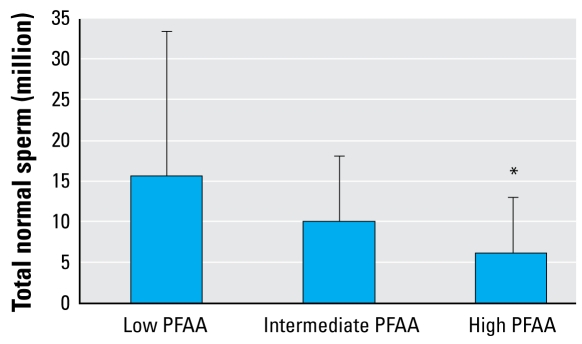
Total morphologically normal spermatozoa (million) adjusted for duration of abstinence and PFAA quartile groups (adjusted mean and 95% confidence interval) in all 105 men. *p= 0.030 compared with low PFAA quartile group.

In the group of all 105 men, we found a tendency toward reduced levels of all semen variables in the high PFAA (PFOS and PFOA) quartile group compared with the low quartile group (Table 2). The difference in percentage of morphologically normal spermatozoa as well as in the total number of normal spermatozoa (total sperm count × percent morphologically normal sperm) was statistically significant (*p* = 0.037 and 0.030, respectively). In the high PFOS–PFOA group, the median number of normal spermatozoa in the ejaculate was 6.2 million compared with 15.5 million in the low group (Figures 1 and 2).

When associations of semen variables to PFOS and PFOA were analyzed separately, as well as the simple summed concentration of PFOS and PFOA, estimated changes in semen variables with a change in serum PFAA concentration of 1 ng/mL indicated negative but nonsignificant associations between the PFAAs and semen variables (Table 3).

### PFAAs and reproductive hormones

We found no significant association between testosterone levels and PFAA levels and no significant difference in PFAA levels between the high and low testosterone groups. For the group of 105 men, adjusted medians for the hormones point to a negative association to PFAA levels; however, none of these tendencies were statistically significant (Table 2).

Estimated associations between reproductive hormones and PFOS and PFOA separately, as well as the summed concentration of PFOS and PFOA, showed no significant associations (Table 4).

### Smoking and BMI

Of the 105 men, 35% were smokers, and there were more smokers in the high testosterone group than in the low testosterone group (49% and 21% smokers, respectively; *p* = 0.003). Smoking status was significantly associated with higher testosterone, lower estradiol, and higher SHBG when entered as a confounder in univariate analyses for these three variables only (*p* = 0.004–0.009). Smoking was not associated with any semen quality variables (*p* = 0.1–0.4) or levels of any PFAA (*p* = 0.1–1.0). Including smoking as a confounder did not considerably change the presented results or significance levels. Therefore, we did not include smoking in the final analyses. BMI was not associated with levels of any PFAA (*p* = 0.1–1.0), or any semen variables (e.g., *p* = 0.5 for morphologically normal sperm, and *p* = 0.9 for total number of morphologically normal sperm in analyses for difference between high and low PFAA groups).

## Discussion

In this study we examined PFAA levels in young adult males. We found high serum concentrations of PFAAs to be significantly associated with reduced numbers of normal spermatozoa. In addition, sperm concentration, total sperm count, and sperm motility showed some tendency toward lower levels in men with high PFAA levels, although not at statistically significant levels. We also found a tendency toward lower inhibin B/FSH ratio with high PFAA levels; this is especially relevant because these hormones reflect spermatogenetic activity.

Testosterone, FAI, testosterone/LH ratio, and testosterone/estradiol ratio could suggest a poorer Leydig cell function in the high-PFAA quartile group compared with the low-PFAA quartile group. However, the associations between reproductive hormones and PFAAs were not completely consistent, and none were significant. Therefore, this study cannot demonstrate an adverse effect of PFAAs on Leydig cell function.

We singled out PFOS and PFOA because no data specifically support PFHxS as an endocrine disruptor, and we divided the men into three groups from the combined concentrations of PFOS and PFOA to account for a potential different effect at the same concentration levels. Because we found no significant association between testosterone levels and PFAA levels and no significant difference in PFAA levels between high and low testosterone groups, we analyzed associations between PFOS–PFOA levels and reproductive hormones or semen variables for the whole group. Controlling for confounding effect of smoking or BMI did not change estimates or significance levels. To our knowledge, there have been no consistent reports of associations between PFAA levels and smoking or BMI.

Our study included only 105 men; in addition, we had selected the men based on their serum testosterone values. The two groups with high and low testosterone were selected in order to test our primary hypothesis, but this selection affects the homogeneity of the group when correlations are analyzed for the group as a whole. This could potentially influence the subsequent analysis of semen quality by bias or confounding and may affect the general applicability of the results. A larger follow-up study would preferably include randomly selected men from the general population.

To our knowledge, the present study is the first to report a correlation between semen quality and PFAAs. Very few studies of other endocrine disruptors (e.g., phthalates, pesticides) have demonstrated such an association (Duty et al. 2003; Hauser et al. 2003, 2007; Meeker et al. 2008; Swan et al. 2003). If the results from our preliminary study of an association between high levels of PFAAs and decreased number of normal sperm are confirmed, then high levels of PFAAs may be regarded as another endocrine-disrupting factor contributing to the low semen quality seen among many young men. In addition, the importance of mixture effects of low-dose exposure to multiple compounds is becoming evident from animal studies but remains to be studied in humans (Hass et al. 2007; Kortenkamp et al. 2007).

The mode of action of PFAAs is not clear. The few animal studies that have explored mechanistic issues show decreased testosterone levels and reduced expression of steroidogenesis genes associated with Leydig cell hyperplasia in adult animals (Biegel et al. 1995; Shi et al. 2007), suggesting a direct testicular effect. Recent studies have indicated that the fetal gonad is particularly sensitive to exogenous factors (Skakkebæk et al. 2001). However, our results could indicate that exposures later in life may contribute to impairment of semen quality, in line with other recent studies (Hauser et al. 2007). We speculate that morphology is perhaps more susceptible to this than sperm concentration or total sperm count. Sperm morphology has proven to be an important indicator of semen quality and fertility in a clinical setting, even in men with normal sperm concentration (Guzick et al. 2001). Interestingly, Fei et al. (2009) reported that higher levels of maternal PFOS and PFOA levels in early pregnancy were associated with significantly longer waiting time to pregnancy. We speculate that men and women living together may have similar exposure to PFAAs and that decreased semen quality caused by high PFAA levels may have contributed to the longer waiting time to pregnancy in that study.

The use and emission of PFCs continue to increase, and PFCs are not readily cleared from the environment (Jensen et al. 2008; Prevedouros et al. 2006). Therefore, humans and wildlife worldwide will be exposed for years to come. We found positive correlations between levels of different PFAAs, as have been reported previously (Apelberg et al. 2007; Calafat et al. 2007; Fei et al. 2007), suggesting common sources of exposure. The PFAA levels we detected are comparable with those found in other countries such as Sweden and the Faroe Islands (Kärrman et al. 2007; Weihe et al. 2008), but lower than results from Denmark from 1996–2002 (Fei et al. 2007). Thus, the effects we found may also apply to other populations.

## Conclusion

Our results indicate that higher PFAA levels are associated with lower numbers of normal sperm. In addition, we found nonsignificant negative associations between PFAA levels and other semen variables and reproductive hormones. Thus, high levels of PFAAs may contribute to the otherwise unexplained low semen quality seen in many young men. However, results from this preliminary study should be corroborated in larger studies.

## Figures and Tables

**Table 1:** Median PFAA concentrations (5th–95th percentiles; ng/mL) for high- and low-testosterone groups and all study subjects.

PFAA	Samples > LOD	High testosterone (*n* = 53)	Low testosterone (*n* = 52)	Whole group (*n* = 105)	*p*-Value
PFHxS	105	6.6 (4.0–13.0)	6.6 (3.5–12.1)	6.6 (4.0–12.1)	0.8
PFHpA	98	0.2 (0.01–0.9)	0.3 (0.00–1.3)	0.2 (0.00–1.1)	0.8
PFOA	105	4.4 (2.6–7.0)	5.0 (2.7–7.5)	4.9 (2.7–7.2)	0.1
PFOS	105	25.5 (14.2–39.6)	23.9 (12.8–45.2)	24.5 (14.2–42.1)	0.4
PFOSA	56	0.1 (0.00–3.7)	0.00 (0.00–3.5)	0.06 (0.00–3.5)	0.008*
PFNA	105	0.8 (0.4–1.8)	0.8 (0.4–2.0)	0.8 (0.4–1.8)	0.6
PFDA	104	0.9 (0.2–1.1)	0.8 (0.4–1.2)	0.9 (0.3–1.1)	0.9
PFUnA	101	0.1 (0.04–0.3)	0.2 (0.00–0.4)	0.1 (0.02– 0.4)	1.0
PFDoA	102	0.08 (0.04–0.8)	0.08 (0.02–0.8)	0.08 (0.04–0.8)	0.8
PFTrA	7	0.00 (0.000–0.4)	0.00 (0.00–0.06)	0.00 (0.00–0.2)	0.2

*p*-Values indicate differences between the high- and low-testosterone groups.

* *p* < 0.05.

**Table 2:** Adjusted mean (95% CI) for PFAA quartile groups (PFOS and PFOA only).

Parameter	Low PFAA (*n* = 29)	Intermediate PFAA (*n* = 48)	High PFAA (*n* = 28)	*p*-Value
Sex hormones^a^				

Testosterone (nmol/L)	25.2 (21.7–28.7)	22.3 (19.6–25.0)	22.3 (18.8–25.8)	0.2
Estradiol (pmol/L)	77.6 (70.3–85.8)	72.4 (67.0–84.7)	76.6 (69.3–84.7)	0.9
SHBG (nmol/L)	27.8 (24.0–32.2)	25.4 (22.6–28.5)	26.1 (22.5–30.3)	0.5
LH (IU/L)	3.4 (2.8–4.1)	2.9 (2.5–3.4)	3.7 (3.0–4.4)	0.6
FSH (IU/L)	2.7 (2.1–3.5)	2.7 (2.2–3.3)	3.0 (2.3–3.8)	0.6
Inhibin-B (pg/mL)	181 (142–232)	175 (144–212)	152 (119–195)	0.3
FAI	84.1 (74.1–95.5)	80.2 (72.6–88.7)	77.8 (68.5–88.3)	0.4
Testosterone/LH	6.9 (5.6–8.4)	7.0 (5.9–8.2)	5.5 (4.5–6.8)	0.1
FAI/LH	24.7 (19.9–30.7)	27.4 (23.1–32.5)	21.2 (17.1–26.4)	0.3
Estradiol/testosterone	3.3 (3.0–3.7)	3.6 (3.2–3.9)	3.8 (3.4–4.2)	0.1
Inhibin/FSH	66.8 (42.1–106.0)	66.0 (45.9–94.8)	51.3 (32.3–81.4)	0.4

Semen quality^b^				

Volume (mL)	4.0 (3.2–5.0)	3.4 (2.9–4.1)	3.5 (2.9–4.4)	0.3
Concentration (million/mL)	59 (36–96)	51 (35–74)	40 (25–64)	0.2
Total count (million)	228 (134–389)	172 (114–261)	143 (86–237)	0.1
Motile sperm (%)	73 (69–77)	70 (66–73)	71 (66–75)	0.4
Morphologically normal (%)	8.8 (7.2–10.4)	7.7 (6.4–9.0)	6.3 (4.6–8.0)	0.037*
Total morphologically normal (million)	15.5 (7.3–33.0)	10.0 (5.6–17.9)	6.23 (3.0–12.8)	0.030*

CI, confidence interval. *p*-Values indicate differences between the high- and low-testosterone groups.

a Hormone levels were adjusted for time of blood sampling.

b Volume, concentration, total count, and total morphologically normal sperm were adjusted for duration of abstinence; motility was adjusted for time between ejaculation and semen analysis; and morphology was not adjusted for confounders.

* *p* < 0.05.

**Table 3:** Estimated change in semen variables^a^ of all 105 men with a change in PFAA of 1 ng/mL (95% CI).

Variable	PFOS	PFOA	PFAA sum^b^
lnSemen volume	0.000 (−0.012 to 0.011)	−0.002 (−0.070 to 0.066)	0.000 (−0.010 to 0.010)
lnSperm concentration	−0.020 (−0.044 to 0.005)	−0.080 (−0.230 to 0.066)	−0.018 (−0.040 to 0.004)
lnTotal sperm count	−0.018 (−0.045 to 0.010)	−0.074 (−0.230 to 0.086)	−0.016 (−0.041 to 0.008)
lnSperm motility	−0.006 (−0.019 to 0.007)	−0.027 (−0.110 to 0.053)	−0.006 (−0.018 to 0.007)
Morphology	−0.085 (−0.200 to 0.026)	−0.540 (−1.200 to 0.110)	−0.082 (−0.181 to 0.018)

CI, confidence interval.

a Volume, concentration, and total count were adjusted for duration of abstinence, motility was adjusted for time between ejaculation and semen analysis, and morphology was not adjusted for confounders.

b Sum of mass concentrations of PFOS and PFOA (ng/mL).

**Table 4:** Estimated change in reproductive hormones^a^ of all 105 men with a change in PFAA concentration of 1 ng/mL (95% CI).

	PFOS	PFOA	PFAA sum^b^
Testosterone	−0.087 (−0.32 to 0.15)	−0.98 (−2.33 to 0.37)	−0.093 (−0.303 to 0.116)
lnEstradiol	−0.001 (−0.008 to 0.005)	−0.012 (−0.051 to 0.027)	−0.001 (−0.007 to 0.005)
lnSHBG	0.002 (−0.007 to 0.012)	−0.009 (−0.067 to 0.048)	0.002 (−0.007 to 0.011)
lnLH	0.000 (−0.014 to 0.012)	−0.010 (−0.084 to 0.064)	0.000 (−0.012 to 0.010)
lnFSH	0.004 (−0.13 to 0.22)	−0.037 (−0.14 to 0.064)	0.003 (−0.013 to 0.018)
lnInhibin B	−0.004 (−0.21 to 0.12)	0.012 (−0.084 to 0.11)	−0.003 (−0.018 to 0.012)
lnFAI	−0.006 (−0.015 to 0.002)	−0.038 (−0.087 to 0.011)	−0.006 (−0.014 to 0.001)
lnTestosterone/LH	−0.003 (−0.017 to 0.011)	−0.037 (−0.12 to 0.045)	−0.003 (−0.016 to 0.009)
lnFAI/LH	−0.006 (−0.020 to 0.009)	−0.028 (−0.114 to 0.058)	−0.005 (−0.018 to 0.008)
lnEstradiol/testosterone	0.003 (−0.005 to 0.010)	0.035 (−0.010 to 0.081)	0.003 (−0.004 to 0.010)
lnInhibin/FSH	−0.009 (−0.039 to 0.022)	0.049 (−0.13 to 0.23)	−0.006 (−0.034 to 0.022)

CI, confidence interval.

a Hormone levels are adjusted for time of blood sampling.

b Sum of mass concentrations of PFOS and PFOA (ng/mL).
